# Complete mitochondrial genome of a parasitoid, *Trichogramma chilonis* (Hymenoptera: Chalcidoidea: Trichogrammatidae) and phylogenetic analysis

**DOI:** 10.1080/23802359.2021.1955636

**Published:** 2021-07-27

**Authors:** Zhi-Ping Xing, Li-Qing Qi, Xu Wang, Long Chen, Ye-Hui Zhu, Yi-Xin Huang, Hao-Yuan Hu

**Affiliations:** a Collaborative Innovation Center of Recovery and Reconstruction of Degraded Ecosystem in Wanjiang Basin Co-founded by Anhui Province and Ministry of Education, School of Ecology and Environment, Anhui Normal University, Wuhu, China; b Anhui Provincial Key Laboratory of the Conservation and Exploitation of Biological Resources, College of Life Sciences, Anhui Normal University, Wuhu, China

**Keywords:** mtDNA, phylogenetic relationship, Trichogrammatidae

## Abstract

*Trichogramma chilonis* is a kind of ovoid parasitic wasp, which has important application value in the biological control of pests. In this study, we sequenced and analyzed the complete mitogenome *T. chilonis* to compare mitogenomic structures and reconstruct phylogenetic relationships. The complete mitogenome sequence of *T. chilonis* is circular, 16,176 bp in size and encodes 13 protein-coding genes (PCGs), 2 ribosomal RNA genes (rRNA), 22 transfer RNA genes (tRNA), and a control region (CR). Nucleotide composition is highly biased toward A + T nucleotides (85.2%). All 13 protein-coding genes (PCGs) initiate with the standard start codon of ATN and terminate with the typical stop codon TAA/TAG. Phylogenetic analyses were performed using amino acids of 13 PCGs showed that *T. chilonis* is closely related to *Trichogramma ostriniae*.

*Trichogramma* (Hymenoptera: Chalcidoidea: Trichogrammatidae) is a kind of oval-parasitic wasp, which can live in the eggs of insect pests (Kuhar et al. 2004). It is widely used in the control of pests in rice, cotton, corn, vegetables, and forest damage (Wang et al. 2004). Thus, *Trichogramma* species are of great value in the biological control of pests.

*Trichogramma chilonis* Ishii, 1941 was collected from the experimental field of Wangwushan Agricultural Science Institute, Jiyuan City, Henan Province, China (116°41′33″, 39°91′09″) and reared until April of 2020, specimens were deposited in the Entomological Museum, College of Life Sciences, Anhui Normal University (YX, Huang, huangyx@ahnu.edu.cn) under the accession no. HN20200430. A whole-genome shotgun (WGS) strategy was used with sequencing on the Illumina Miseq platform. Raw data were retrieved and qualified by FastQC (http://www.bioinformatics.babraham.ac.uk/projects/fastqc). The raw paired reads were quality-trimmed and assembled into the complete circular mitogenome in Geneious v 11.0.2 with default parameters (Kearse et al. 2012). Protein-coding genes (PCGs) were predicted as open reading frames (ORFs) corresponding to 13 PCGs in invertebrate mitochondrial genetic code.

The genome sequence data that support the findings of this study are openly available in GenBank of NCBI at (https://www.ncbi.nlm.nih.gov/) under the accession no. MT712144). The complete mitogenome length of *T. chilonis* is 16,176 bp, circular and double-stranded. It is composed of the typical 37 mitochondrial genes, including 13 PCGs, 22 tRNAs, 2 rRNAs, and a control region (Cameron 2014). The composition of *T. chilonis* is similar to many insect mitogenomes reported previously. The majority strand (J-strand) encodes 31 genes (11 PCGs,18 tRNAs, and 2 rRNAs), and the minority strand (N-strand) encodes 6 genes (2 PCGs and 4 tRNAs). The overall base composition of the mitogenome was estimated to be A: 47.0%, T: 38.2%, C: 8.5%, and G: 6.3%, with a high AT content of 85.2%. All 13 PCGs are composed of seven NADH dehydrogenase subunits, three cytochrome c oxidase subunits, two ATPase subunits, and one cytochrome b gene. Among the 13 PCGs in the *T. chilonis* mitogenome, 11 PCGs (*nad1, nad2, nad3, nad4, nad4l, nad5, cox1, cox2, cox3, atp6, atp8*) are encoded by the J strand, while the other two PCGs are encoded on the N strand. All 13 PCGs start with ATN and stop with traditional TAA or TAG codons, which is similar to most other insect mitogenomes (Crozier and Crozier 1993; Korkmaz et al. 2015).

To validate the phylogenetic position of *T. chilonis*, we selected the mitochondrial DNA sequences of 38 Hymenoptera species (36 Chalcidoidea as ingroup and remainder as outgroup). Amino acids sequences from each PCG were aligned by MAFFT (Katoh et al. 2005). Then concatenate the aligned sequences into a dataset. Phylogenetic trees were reconstructed by using the maximum-likelihood method (ML) on the W-IQ-Tree webserver (Trifinopoulos et al. 2016). The result showed that all the families formed monophyletic groups respectively (Figure 1). *Trichogramma chilonis* is closely related to *Trichogramma ostriniae*. *Megaphragam* formed a sister group with *Trichogramma*, which is in accordance with the previous study (Chen et al. 2018).

**Figure 1.**
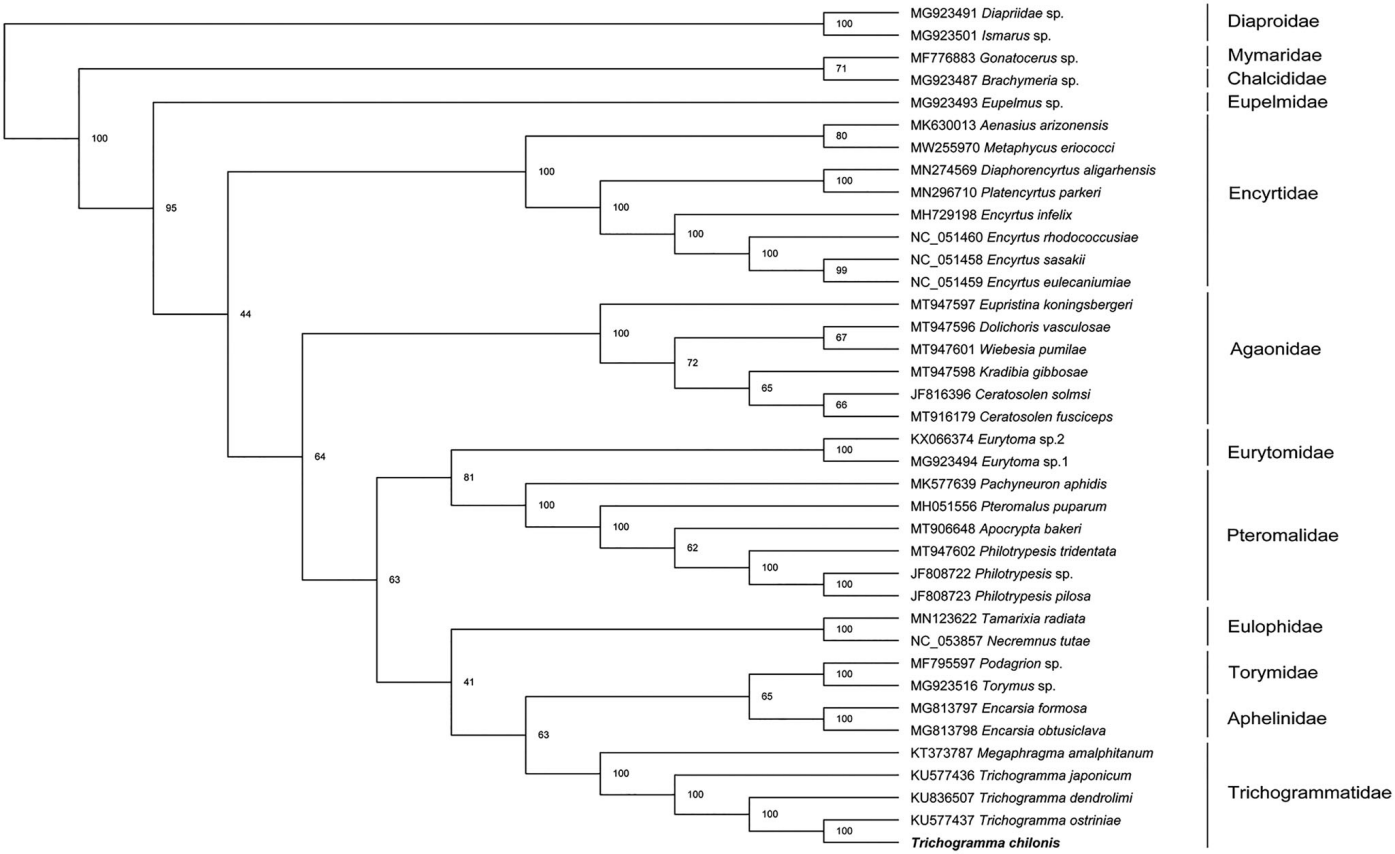
Phylogenetic relationships within Chalcidoidea based on the amino acid sequences of 13 protein-coding genes were performed using ML methods.

## Data Availability

The data that support the findings of this study are openly available in GenBank at https://www.ncbi.nlm.nih.gov/genbank/, reference number MT712144. The associated BioProject, Bio-Sample numbers, and SRA are PRJNA727057, SAMN19004953, and SRR14416419, respectively.

## References

[CIT0001] Cameron SL. 2014. Insect mitochondrial genomics: implications for evolution and phylogeny. Annu Rev Entomol. 59:95–117. 24160435 10.1146/annurev-ento-011613-162007

[CIT0002] Chen L, Chen PY, Xue XF, Hua HQ, Li YX, Zhang F, Wei SJ. 2018. Extensive gene rearrangements in the mitochondrial genomes of two egg parasitoids, *Trichogramma japonicum* and *Trichogramma ostriniae* (Hymenoptera: Chalcidoidea: Trichogrammatidae). Sci Rep. 8(1):7034. 29728615 10.1038/s41598-018-25338-3 PMC5935716

[CIT0003] Crozier RH, Crozier YC. 1993. The mitochondrial genome of the honeybee *Apis mellifera*: complete sequence and genome organization. Genetics. 133(1):97–117. 8417993 10.1093/genetics/133.1.97 PMC1205303

[CIT0004] Katoh K, Kuma KI, Toh H, Miyata T. 2005. MAFFT version 5: improvement in accuracy of multiple sequence alignment. Nucleic Acids Res. 33(2):511–518. 15661851 10.1093/nar/gki198 PMC548345

[CIT0005] Kearse M, Moir R, Wilson A, Stones-Havas S, Cheung M, Sturrock S, Buxton S, Cooper A, Markowitz S, Duran C, et al. 2012. Geneious basic: an integrated and extendable desktop software platform for the organization and analysis of sequence data. Bioinformatics. 28(12):1647–1649. 22543367 10.1093/bioinformatics/bts199 PMC3371832

[CIT0006] Korkmaz EM, Doğan Ö, Budak M, Başıbüyük HH. 2015. Two nearly complete mitogenomes of wheat stem borers*, Cephus pygmeus* (L.) and *Cephus sareptanus* Dovnar-Zapolskij (Hymenoptera: Cephidae): an unusual elongation of *rrnS* gene. Gene. 558(2):254–264. 25576223 10.1016/j.gene.2014.12.069

[CIT0007] Kuhar TP, Barlow VM, Hoffmann MP, Fleischer SJ, Groden E, Gardner J, Hazzard R, Wright MG, Pitcher SA, Speese J, III, et al. 2004. Potential of *Trichogramma ostriniae* (Hymenoptera: Trichogrammatidae) for biological control of European corn borer (Lepidoptera: Crambidae) in solanaceous crops. J Econ Entomol. 97(4):1209–1216. 15384329 10.1093/jee/97.4.1209

[CIT0008] Trifinopoulos J, Nguyen LT, Haeseler AV, Minh BQ. 2016. W-IQ-TREE: a fast online phylogenetic tool for maximum likelihood analysis. Nucleic Acids Res. 44(W1):W232–W235. 27084950 10.1093/nar/gkw256 PMC4987875

[CIT0009] Wang B, Ferro DN, Wu J, Wang SQ. 2004. Temperature-dependent development and oviposition behavior of *Trichogramma ostriniae* (Hymenoptera: Trichogrammatidae), a potential biological control agent for the European corn borer (Lepidoptera: Crambidae). Environ Entomol. 33(4):787–793.

